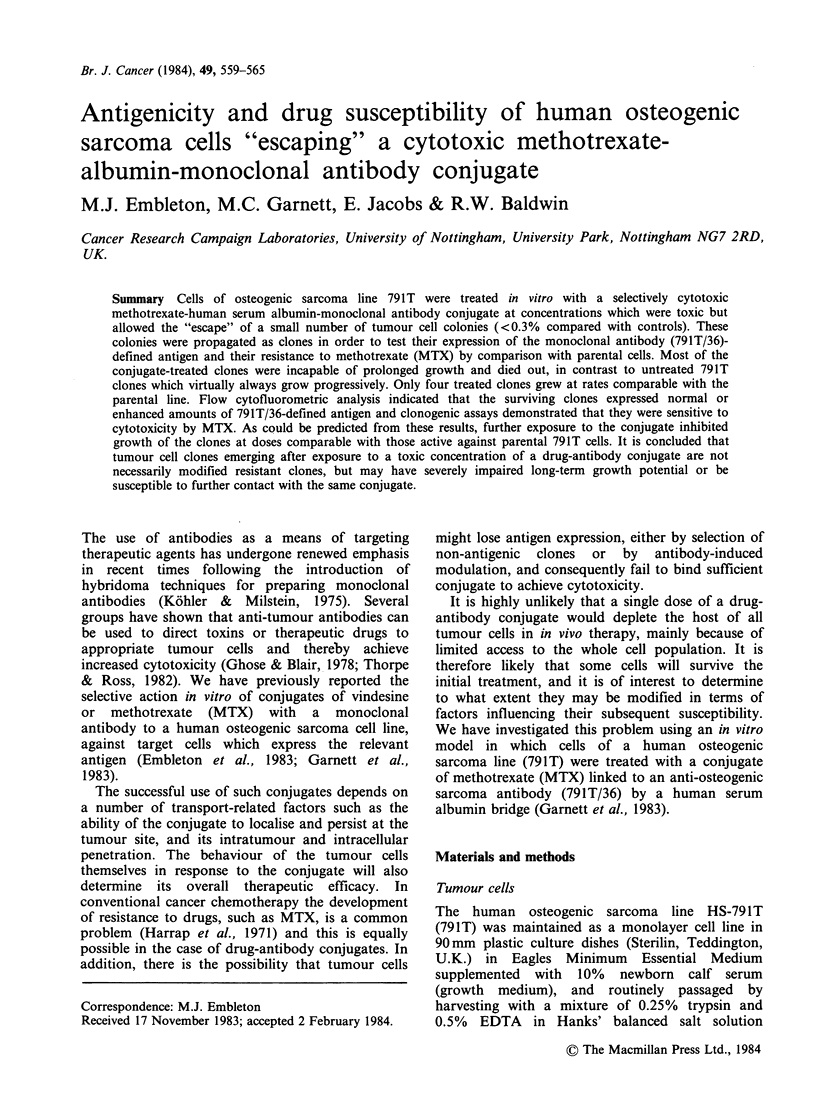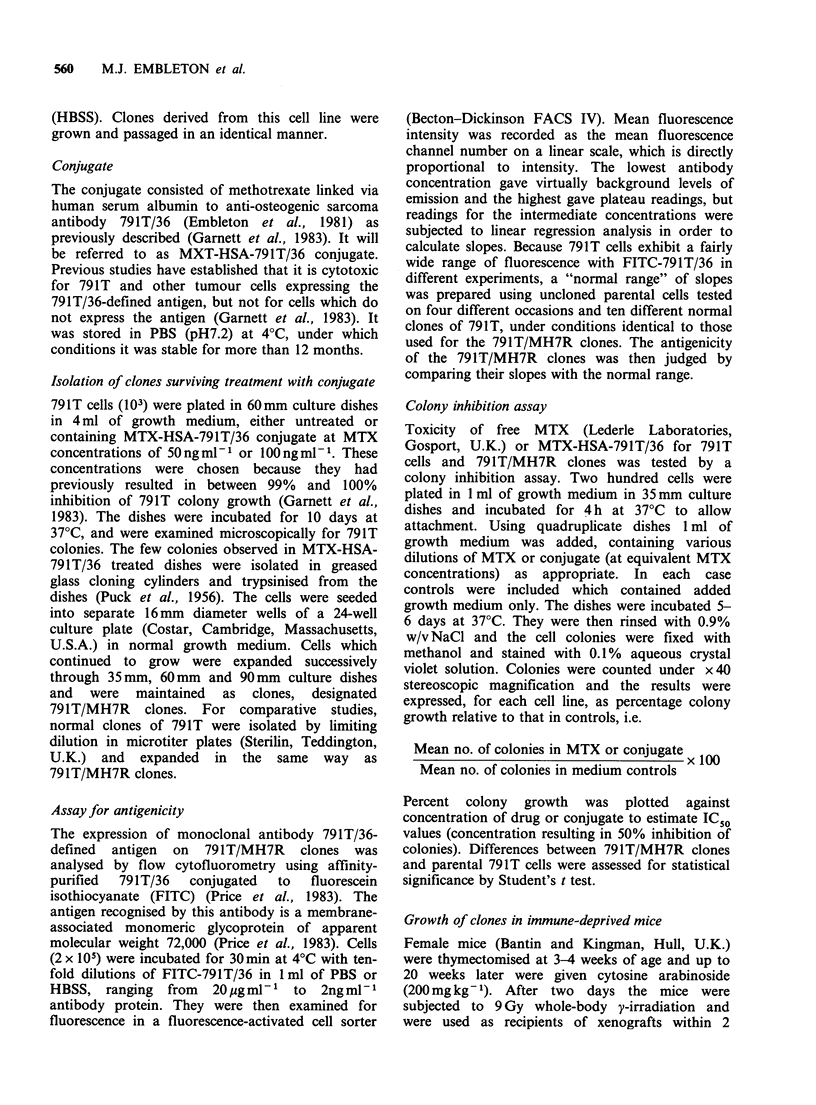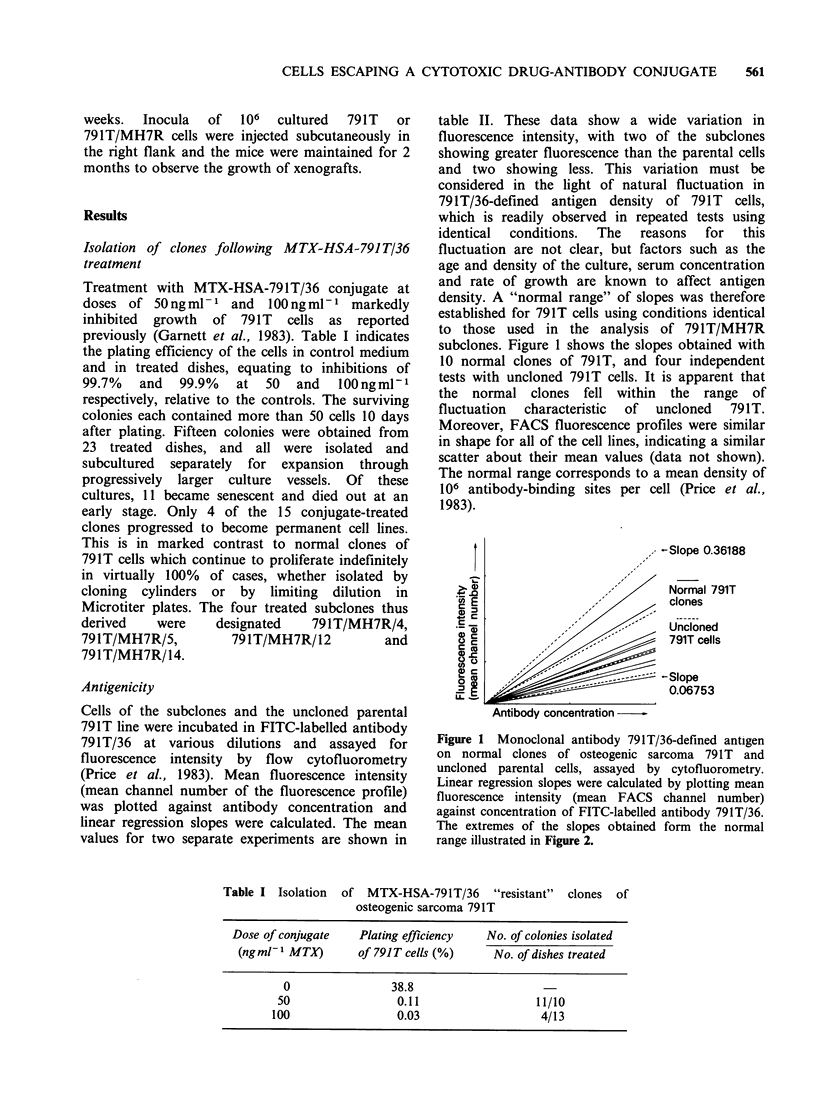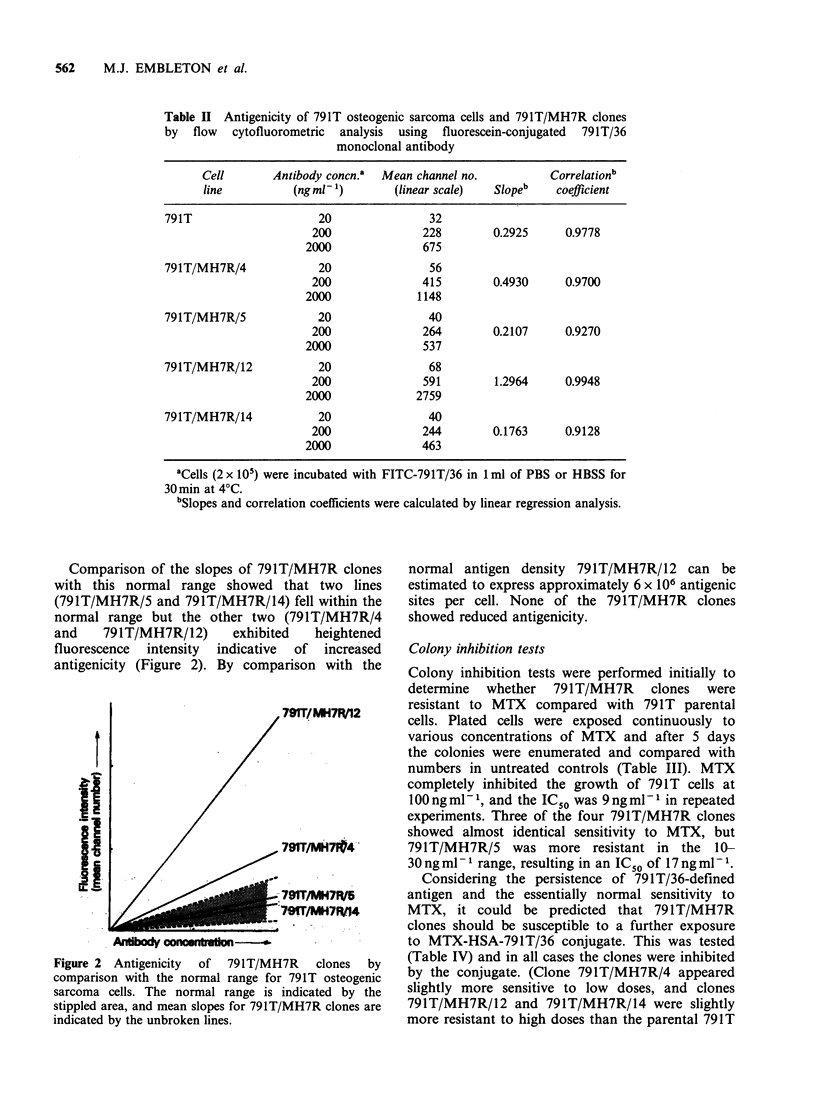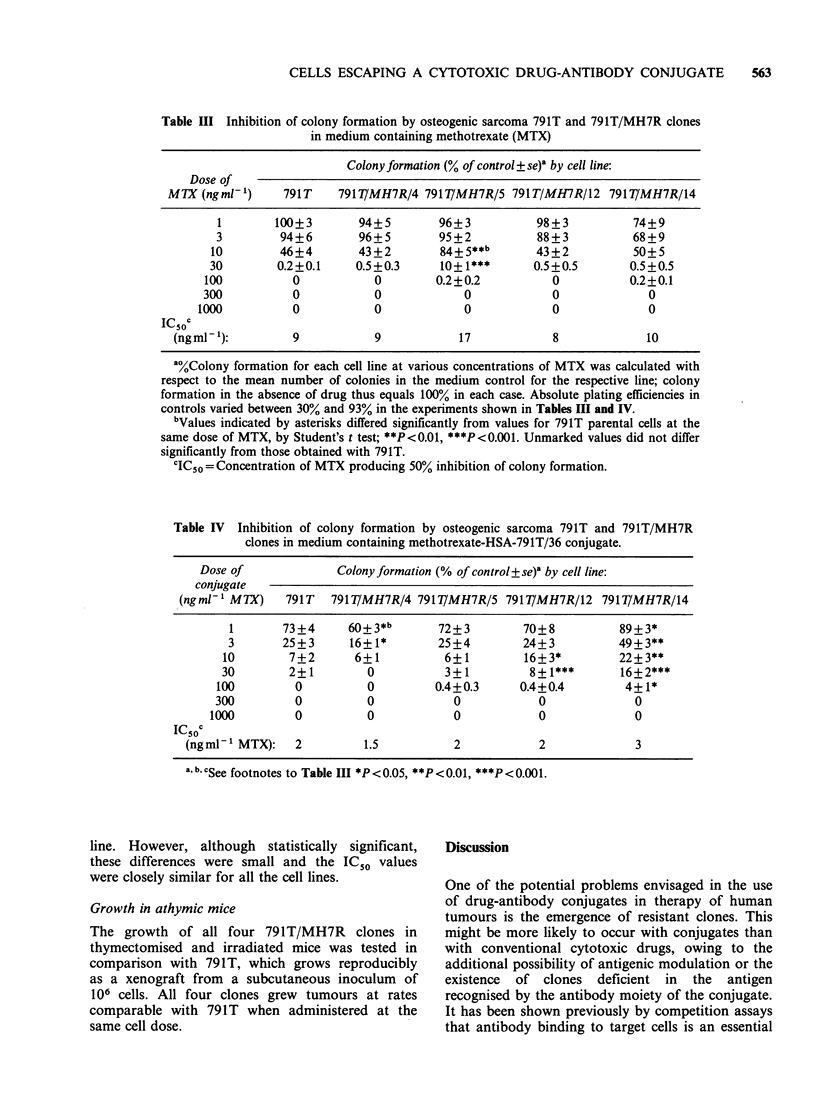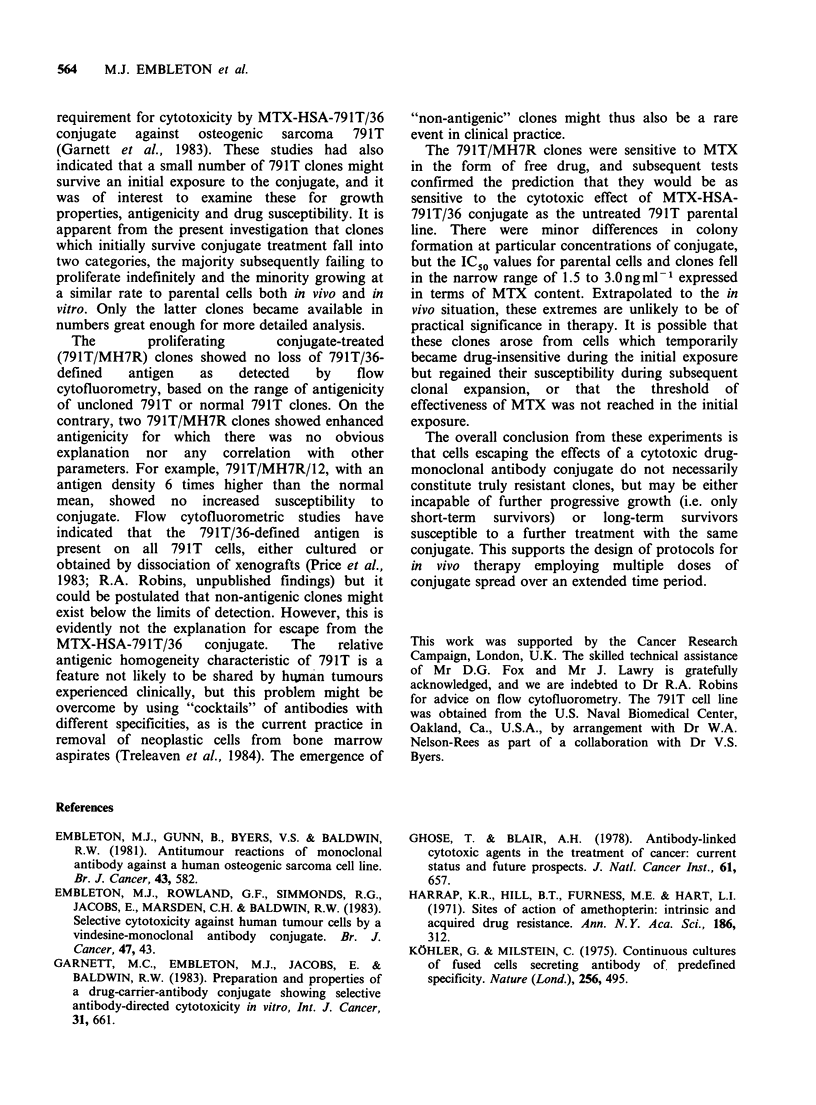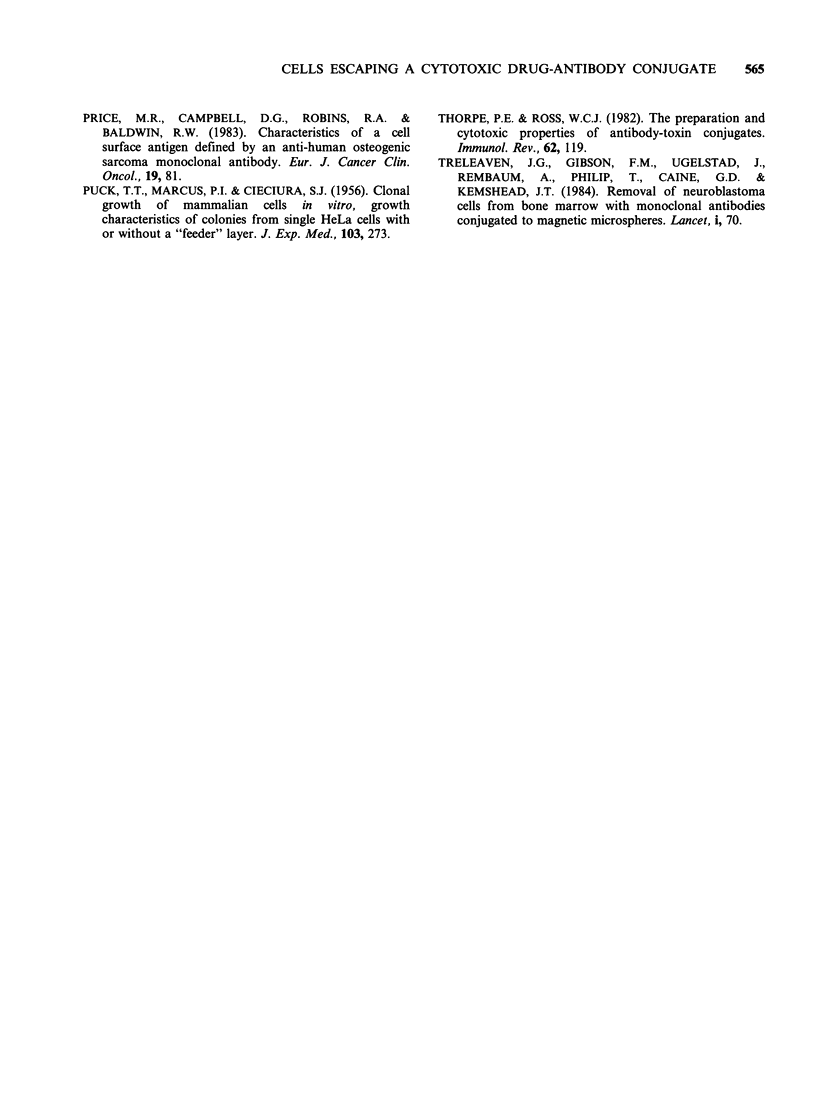# Antigenicity and drug susceptibility of human osteogenic sarcoma cells "escaping" a cytotoxic methotrexate-albumin-monoclonal antibody conjugate.

**DOI:** 10.1038/bjc.1984.89

**Published:** 1984-05

**Authors:** M. J. Embleton, M. C. Garnett, E. Jacobs, R. W. Baldwin

## Abstract

Cells of osteogenic sarcoma line 791T were treated in vitro with a selectively cytotoxic methotrexate-human serum albumin-monoclonal antibody conjugate at concentrations which were toxic but allowed the "escape" of a small number of tumour cell colonies (less than 0.3% compared with controls). These colonies were propagated as clones in order to test their expression of the monoclonal antibody ( 791T /36)-defined antigen and their resistance to methotrexate (MTX) by comparison with parental cells. Most of the conjugate-treated clones were incapable of prolonged growth and died out, in contrast to untreated 791T clones which virtually always grow progressively. Only four treated clones grew at rates comparable with the parental line. Flow cytofluorometric analysis indicated that the surviving clones expressed normal or enhanced amounts of 791T /36-defined antigen and clonogenic assays demonstrated that they were sensitive to cytotoxicity by MTX. As could be predicted from these results, further exposure to the conjugate inhibited growth of the clones at doses comparable with those active against parental 791T cells. It is concluded that tumour cell clones emerging after exposure to a toxic concentration of a drug-antibody conjugate are not necessarily modified resistant clones, but may have severely impaired long-term growth potential or be susceptible to further contact with the same conjugate.


*Br. J. Cancer* (1984), **49**, 559–565

### Antigenicity and drug susceptibility of human osteogenic sarcoma cells "escaping" a cytotoxic methotrexate-albumin-monoclonal antibody conjugate

M.J. Embleton, M.C. Garnett, E. Jacobs & R.W. Baldwin

*Cancer Research Campaign Laboratories, University of Nottingham, University Park, Nottingham NG7 2RD, UK.*

**Summary** Cells of osteogenic sarcoma line 791T were treated *in vitro* with a selectively cytotoxic methotrexate-human serum albumin-monoclonal antibody conjugate at concentrations which were toxic but allowed the "escape" of a small number of tumour cell colonies (<0.3% compared with controls). These colonies were propagated as clones in order to test their expression of the monoclonal antibody (791T/36)-defined antigen and their resistance to methotrexate (MTX) by comparison with parental cells. Most of the conjugate-treated clones were incapable of prolonged growth and died out, in contrast to untreated 791T clones which virtually always grow progressively. Only four treated clones grew at rates comparable with the parental line. Flow cytofluorometric analysis indicated that the surviving clones expressed normal or enhanced amounts of 791T/36-defined antigen and clonogenic assays demonstrated that they were sensitive to cytotoxicity by MTX. As could be predicted from these results, further exposure to the conjugate inhibited growth of the clones at doses comparable with those active against parental 791T cells. It is concluded that tumour cell clones emerging after exposure to a toxic concentration of a drug-antibody conjugate are not necessarily modified resistant clones, but may have severely impaired long-term growth potential or be susceptible to further contact with the same conjugate.

The use of antibodies as a means of targeting therapeutic agents has undergone renewed emphasis in recent times following the introduction of hybridoma techniques for preparing monoclonal antibodies (Köhler & Milstein, 1975). Several groups have shown that anti-tumour antibodies can be used to direct toxins or therapeutic drugs to appropriate tumour cells and thereby achieve increased cytotoxicity (Ghose & Blair, 1978; Thorpe & Ross, 1982). We have previously reported the selective action *in vitro* of conjugates of vindesine or methotrexate (MTX) with a monoclonal antibody to a human osteogenic sarcoma cell line, against target cells which express the relevant antigen (Embleton *et al.*, 1983; Garnett *et al.*, 1983).

The successful use of such conjugates depends on a number of transport-related factors such as the ability of the conjugate to localise and persist at the tumour site, and its intratumour and intracellular penetration. The behaviour of the tumour cells themselves in response to the conjugate will also determine its overall therapeutic efficacy. In conventional cancer chemotherapy the development of resistance to drugs, such as MTX, is a common problem (Harrap *et al.*, 1971) and this is equally possible in the case of drug-antibody conjugates. In addition, there is the possibility that tumour cells might lose antigen expression, either by selection of non-antigenic clones or by antibody-induced modulation, and consequently fail to bind sufficient conjugate to achieve cytotoxicity.

It is highly unlikely that a single dose of a drug-antibody conjugate would deplete the host of all tumour cells in *in vivo* therapy, mainly because of limited access to the whole cell population. It is therefore likely that some cells will survive the initial treatment, and it is of interest to determine to what extent they may be modified in terms of factors influencing their subsequent susceptibility. We have investigated this problem using an *in vitro* model in which cells of a human osteogenic sarcoma line (791T) were treated with a conjugate of methotrexate (MTX) linked to an anti-osteogenic sarcoma antibody (791T/36) by a human serum albumin bridge (Garnett *et al.*, 1983).

### Materials and methods

### *Tumour cells*

The human osteogenic sarcoma line HS-791T (791T) was maintained as a monolayer cell line in 90 mm plastic culture dishes (Sterilin, Teddington, U.K.) in Eagles Minimum Essential Medium supplemented with 10% newborn calf serum (growth medium), and routinely passaged by harvesting with a mixture of 0.25% trypsin and 0.5% EDTA in Hanks' balanced salt solution

Correspondence: M.J. Embleton
Received 17 November 1983; accepted 2 February 1984.

© The Macmillan Press Ltd., 1984

(HBSS). Clones derived from this cell line were grown and passaged in an identical manner.

*Conjugate*

The conjugate consisted of methotrexate linked via human serum albumin to anti-osteogenic sarcoma antibody 791T/36 (Embleton *et al.*, 1981) as previously described (Garnett *et al.*, 1983). It will be referred to as MXT-HSA-791T/36 conjugate. Previous studies have established that it is cytotoxic for 791T and other tumour cells expressing the 791T/36-defined antigen, but not for cells which do not express the antigen (Garnett *et al.*, 1983). It was stored in PBS (pH7.2) at 4°C, under which conditions it was stable for more than 12 months.

*Isolation of clones surviving treatment with conjugate*

791T cells ($10^3$) were plated in 60 mm culture dishes in 4 ml of growth medium, either untreated or containing MTX-HSA-791T/36 conjugate at MTX concentrations of 50 ng ml$^{-1}$ or 100 ng ml$^{-1}$. These concentrations were chosen because they had previously resulted in between 99% and 100% inhibition of 791T colony growth (Garnett *et al.*, 1983). The dishes were incubated for 10 days at 37°C, and were examined microscopically for 791T colonies. The few colonies observed in MTX-HSA-791T/36 treated dishes were isolated in greased glass cloning cylinders and trypsinised from the dishes (Puck *et al.*, 1956). The cells were seeded into separate 16 mm diameter wells of a 24-well culture plate (Costar, Cambridge, Massachusetts, U.S.A.) in normal growth medium. Cells which continued to grow were expanded successively through 35 mm, 60 mm and 90 mm culture dishes and were maintained as clones, designated 791T/MH7R clones. For comparative studies, normal clones of 791T were isolated by limiting dilution in microtiter plates (Sterilin, Teddington, U.K.) and expanded in the same way as 791T/MH7R clones.

*Assay for antigenicity*

The expression of monoclonal antibody 791T/36-defined antigen on 791T/MH7R clones was analysed by flow cytofluorometry using affinity-purified 791T/36 conjugated to fluorescein isothiocyanate (FITC) (Price *et al.*, 1983). The antigen recognised by this antibody is a membrane-associated monomeric glycoprotein of apparent molecular weight 72,000 (Price *et al.*, 1983). Cells ($2 \times 10^5$) were incubated for 30 min at 4°C with ten-fold dilutions of FITC-791T/36 in 1 ml of PBS or HBSS, ranging from 20 $\mu$g ml$^{-1}$ to 2 ng ml$^{-1}$ antibody protein. They were then examined for fluorescence in a fluorescence-activated cell sorter (Becton–Dickinson FACS IV). Mean fluorescence intensity was recorded as the mean fluorescence channel number on a linear scale, which is directly proportional to intensity. The lowest antibody concentration gave virtually background levels of emission and the highest gave plateau readings, but readings for the intermediate concentrations were subjected to linear regression analysis in order to calculate slopes. Because 791T cells exhibit a fairly wide range of fluorescence with FITC-791T/36 in different experiments, a "normal range" of slopes was prepared using uncloned parental cells tested on four different occasions and ten different normal clones of 791T, under conditions identical to those used for the 791T/MH7R clones. The antigenicity of the 791T/MH7R clones was then judged by comparing their slopes with the normal range.

*Colony inhibition assay*

Toxicity of free MTX (Lederle Laboratories, Gosport, U.K.) or MTX-HSA-791T/36 for 791T cells and 791T/MH7R clones was tested by a colony inhibition assay. Two hundred cells were plated in 1 ml of growth medium in 35 mm culture dishes and incubated for 4 h at 37°C to allow attachment. Using quadruplicate dishes 1 ml of growth medium was added, containing various dilutions of MTX or conjugate (at equivalent MTX concentrations) as appropriate. In each case controls were included which contained added growth medium only. The dishes were incubated 5–6 days at 37°C. They were then rinsed with 0.9% w/v NaCl and the cell colonies were fixed with methanol and stained with 0.1% aqueous crystal violet solution. Colonies were counted under $\times 40$ stereoscopic magnification and the results were expressed, for each cell line, as percentage colony growth relative to that in controls, i.e.

$$\frac{\text{Mean no. of colonies in MTX or conjugate}}{\text{Mean no. of colonies in medium controls}} \times 100$$

Percent colony growth was plotted against concentration of drug or conjugate to estimate $IC_{50}$ values (concentration resulting in 50% inhibition of colonies). Differences between 791T/MH7R clones and parental 791T cells were assessed for statistical significance by Student's $t$ test.

*Growth of clones in immune-deprived mice*

Female mice (Bantin and Kingman, Hull, U.K.) were thymectomised at 3–4 weeks of age and up to 20 weeks later were given cytosine arabinoside (200 mg kg$^{-1}$). After two days the mice were subjected to 9 Gy whole-body $\gamma$-irradiation and were used as recipients of xenografts within 2

weeks. Inocula of $10^6$ cultured 791T or 791T/MH7R cells were injected subcutaneously in the right flank and the mice were maintained for 2 months to observe the growth of xenografts.

### Results

### *Isolation of clones following MTX-HSA-791T/36 treatment*

Treatment with MTX-HSA-791T/36 conjugate at doses of $50\,ng\,ml^{-1}$ and $100\,ng\,ml^{-1}$ markedly inhibited growth of 791T cells as reported previously (Garnett *et al.*, 1983). Table I indicates the plating efficiency of the cells in control medium and in treated dishes, equating to inhibitions of 99.7% and 99.9% at 50 and $100\,ng\,ml^{-1}$ respectively, relative to the controls. The surviving colonies each contained more than 50 cells 10 days after plating. Fifteen colonies were obtained from 23 treated dishes, and all were isolated and subcultured separately for expansion through progressively larger culture vessels. Of these cultures, 11 became senescent and died out at an early stage. Only 4 of the 15 conjugate-treated clones progressed to become permanent cell lines. This is in marked contrast to normal clones of 791T cells which continue to proliferate indefinitely in virtually 100% of cases, whether isolated by cloning cylinders or by limiting dilution in Microtiter plates. The four treated subclones thus derived were designated 791T/MH7R/4, 791T/MH7R/5, 791T/MH7R/12 and 791T/MH7R/14.

### *Antigenicity*

Cells of the subclones and the uncloned parental 791T line were incubated in FITC-labelled antibody 791T/36 at various dilutions and assayed for fluorescence intensity by flow cytofluorometry (Price *et al.*, 1983). Mean fluorescence intensity (mean channel number of the fluorescence profile) was plotted against antibody concentration and linear regression slopes were calculated. The mean values for two separate experiments are shown in table II. These data show a wide variation in fluorescence intensity, with two of the subclones showing greater fluorescence than the parental cells and two showing less. This variation must be considered in the light of natural fluctuation in 791T/36-defined antigen density of 791T cells, which is readily observed in repeated tests using identical conditions. The reasons for this fluctuation are not clear, but factors such as the age and density of the culture, serum concentration and rate of growth are known to affect antigen density. A "normal range" of slopes was therefore established for 791T cells using conditions identical to those used in the analysis of 791T/MH7R subclones. Figure 1 shows the slopes obtained with 10 normal clones of 791T, and four independent tests with uncloned 791T cells. It is apparent that the normal clones fell within the range of fluctuation characteristic of uncloned 791T. Moreover, FACS fluorescence profiles were similar in shape for all of the cell lines, indicating a similar scatter about their mean values (data not shown). The normal range corresponds to a mean density of $10^6$ antibody-binding sites per cell (Price *et al.*, 1983).

**Figure 1** Monoclonal antibody 791T/36-defined antigen on normal clones of osteogenic sarcoma 791T and uncloned parental cells, assayed by cytofluorometry. Linear regression slopes were calculated by plotting mean fluorescence intensity (mean FACS channel number) against concentration of FITC-labelled antibody 791T/36. The extremes of the slopes obtained form the normal range illustrated in **Figure 2**.

**Table I** Isolation of MTX-HSA-791T/36 "resistant" clones of osteogenic sarcoma 791T

| *Dose of conjugate* (*ng ml*$^{-1}$ *MTX*) | *Plating efficiency of 791T cells* (%) | *No. of colonies isolated* / *No. of dishes treated* |
|---|---|---|
| 0 | 38.8 | — |
| 50 | 0.11 | 11/10 |
| 100 | 0.03 | 4/13 |

**Table II** Antigenicity of 791T osteogenic sarcoma cells and 791T/MH7R clones by flow cytofluorometric analysis using fluorescein-conjugated 791T/36 monoclonal antibody

| *Cell line* | *Antibody concn.*[a] *(ng ml$^{-1}$)* | *Mean channel no. (linear scale)* | *Slope*[b] | *Correlation*[b] *coefficient* |
|---|---|---|---|---|
| 791T | 20 | 32 | | |
| | 200 | 228 | 0.2925 | 0.9778 |
| | 2000 | 675 | | |
| 791T/MH7R/4 | 20 | 56 | | |
| | 200 | 415 | 0.4930 | 0.9700 |
| | 2000 | 1148 | | |
| 791T/MH7R/5 | 20 | 40 | | |
| | 200 | 264 | 0.2107 | 0.9270 |
| | 2000 | 537 | | |
| 791T/MH7R/12 | 20 | 68 | | |
| | 200 | 591 | 1.2964 | 0.9948 |
| | 2000 | 2759 | | |
| 791T/MH7R/14 | 20 | 40 | | |
| | 200 | 244 | 0.1763 | 0.9128 |
| | 2000 | 463 | | |

[a]Cells ($2 \times 10^5$) were incubated with FITC-791T/36 in 1 ml of PBS or HBSS for 30 min at 4°C.

[b]Slopes and correlation coefficients were calculated by linear regression analysis.

Comparison of the slopes of 791T/MH7R clones with this normal range showed that two lines (791T/MH7R/5 and 791T/MH7R/14) fell within the normal range but the other two (791T/MH7R/4 and 791T/MH7R/12) exhibited heightened fluorescence intensity indicative of increased antigenicity (Figure 2). By comparison with the

**Figure 2** Antigenicity of 791T/MH7R clones by comparison with the normal range for 791T osteogenic sarcoma cells. The normal range is indicated by the stippled area, and mean slopes for 791T/MH7R clones are indicated by the unbroken lines.

normal antigen density 791T/MH7R/12 can be estimated to express approximately $6 \times 10^6$ antigenic sites per cell. None of the 791T/MH7R clones showed reduced antigenicity.

### *Colony inhibition tests*

Colony inhibition tests were performed initially to determine whether 791T/MH7R clones were resistant to MTX compared with 791T parental cells. Plated cells were exposed continuously to various concentrations of MTX and after 5 days the colonies were enumerated and compared with numbers in untreated controls (Table III). MTX completely inhibited the growth of 791T cells at 100 ng ml$^{-1}$, and the $IC_{50}$ was 9 ng ml$^{-1}$ in repeated experiments. Three of the four 791T/MH7R clones showed almost identical sensitivity to MTX, but 791T/MH7R/5 was more resistant in the 10–30 ng ml$^{-1}$ range, resulting in an $IC_{50}$ of 17 ng ml$^{-1}$.

Considering the persistence of 791T/36-defined antigen and the essentially normal sensitivity to MTX, it could be predicted that 791T/MH7R clones should be susceptible to a further exposure to MTX-HSA-791T/36 conjugate. This was tested (Table IV) and in all cases the clones were inhibited by the conjugate. (Clone 791T/MH7R/4 appeared slightly more sensitive to low doses, and clones 791T/MH7R/12 and 791T/MH7R/14 were slightly more resistant to high doses than the parental 791T

**Table III** Inhibition of colony formation by osteogenic sarcoma 791T and 791T/MH7R clones in medium containing methotrexate (MTX)

| *Dose of MTX* ($ng\,ml^{-1}$) | *Colony formation (% of control ± se)*[a] *by cell line:* | | | | |
|---|---|---|---|---|---|
| | *791T* | *791T/MH7R/4* | *791T/MH7R/5* | *791T/MH7R/12* | *791T/MH7R/14* |
| 1 | 100±3 | 94±5 | 96±3 | 98±3 | 74±9 |
| 3 | 94±6 | 96±5 | 95±2 | 88±3 | 68±9 |
| 10 | 46±4 | 43±2 | 84±5**[b] | 43±2 | 50±5 |
| 30 | 0.2±0.1 | 0.5±0.3 | 10±1*** | 0.5±0.5 | 0.5±0.5 |
| 100 | 0 | 0 | 0.2±0.2 | 0 | 0.2±0.1 |
| 300 | 0 | 0 | 0 | 0 | 0 |
| 1000 | 0 | 0 | 0 | 0 | 0 |
| $IC_{50}$[c] ($ng\,ml^{-1}$): | 9 | 9 | 17 | 8 | 10 |

[a]%Colony formation for each cell line at various concentrations of MTX was calculated with respect to the mean number of colonies in the medium control for the respective line; colony formation in the absence of drug thus equals 100% in each case. Absolute plating efficiencies in controls varied between 30% and 93% in the experiments shown in **Tables III and IV**.

[b]Values indicated by asterisks differed significantly from values for 791T parental cells at the same dose of MTX, by Student's *t* test; $**P<0.01$, $***P<0.001$. Unmarked values did not differ significantly from those obtained with 791T.

[c]$IC_{50}$ = Concentration of MTX producing 50% inhibition of colony formation.

**Table IV** Inhibition of colony formation by osteogenic sarcoma 791T and 791T/MH7R clones in medium containing methotrexate-HSA-791T/36 conjugate.

| *Dose of conjugate* ($ng\,ml^{-1}$ *MTX*) | *Colony formation (% of control ± se)*[a] *by cell line:* | | | | |
|---|---|---|---|---|---|
| | *791T* | *791T/MH7R/4* | *791T/MH7R/5* | *791T/MH7R/12* | *791T/MH7R/14* |
| 1 | 73±4 | 60±3*[b] | 72±3 | 70±8 | 89±3* |
| 3 | 25±3 | 16±1* | 25±4 | 24±3 | 49±3** |
| 10 | 7±2 | 6±1 | 6±1 | 16±3* | 22±3** |
| 30 | 2±1 | 0 | 3±1 | 8±1*** | 16±2*** |
| 100 | 0 | 0 | 0.4±0.3 | 0.4±0.4 | 4±1* |
| 300 | 0 | 0 | 0 | 0 | 0 |
| 1000 | 0 | 0 | 0 | 0 | 0 |
| $IC_{50}$[c] ($ng\,ml^{-1}$ MTX): | 2 | 1.5 | 2 | 2 | 3 |

[a, b, c]See footnotes to **Table III** $*P<0.05$, $**P<0.01$, $***P<0.001$.

line. However, although statistically significant, these differences were small and the $IC_{50}$ values were closely similar for all the cell lines.

### *Growth in athymic mice*

The growth of all four 791T/MH7R clones in thymectomised and irradiated mice was tested in comparison with 791T, which grows reproducibly as a xenograft from a subcutaneous inoculum of $10^6$ cells. All four clones grew tumours at rates comparable with 791T when administered at the same cell dose.

### Discussion

One of the potential problems envisaged in the use of drug-antibody conjugates in therapy of human tumours is the emergence of resistant clones. This might be more likely to occur with conjugates than with conventional cytotoxic drugs, owing to the additional possibility of antigenic modulation or the existence of clones deficient in the antigen recognised by the antibody moiety of the conjugate. It has been shown previously by competition assays that antibody binding to target cells is an essential requirement for cytotoxicity by MTX-HSA-791T/36 conjugate against osteogenic sarcoma 791T (Garnett *et al.*, 1983). These studies had also indicated that a small number of 791T clones might survive an initial exposure to the conjugate, and it was of interest to examine these for growth properties, antigenicity and drug susceptibility. It is apparent from the present investigation that clones which initially survive conjugate treatment fall into two categories, the majority subsequently failing to proliferate indefinitely and the minority growing at a similar rate to parental cells both *in vivo* and *in vitro*. Only the latter clones became available in numbers great enough for more detailed analysis.

The proliferating conjugate-treated (791T/MH7R) clones showed no loss of 791T/36-defined antigen as detected by flow cytofluorometry, based on the range of antigenicity of uncloned 791T or normal 791T clones. On the contrary, two 791T/MH7R clones showed enhanced antigenicity for which there was no obvious explanation nor any correlation with other parameters. For example, 791T/MH7R/12, with an antigen density 6 times higher than the normal mean, showed no increased susceptibility to conjugate. Flow cytofluorometric studies have indicated that the 791T/36-defined antigen is present on all 791T cells, either cultured or obtained by dissociation of xenografts (Price *et al.*, 1983; R.A. Robins, unpublished findings) but it could be postulated that non-antigenic clones might exist below the limits of detection. However, this is evidently not the explanation for escape from the MTX-HSA-791T/36 conjugate. The relative antigenic homogeneity characteristic of 791T is a feature not likely to be shared by human tumours experienced clinically, but this problem might be overcome by using "cocktails" of antibodies with different specificities, as is the current practice in removal of neoplastic cells from bone marrow aspirates (Treleaven *et al.*, 1984). The emergence of "non-antigenic" clones might thus also be a rare event in clinical practice.

The 791T/MH7R clones were sensitive to MTX in the form of free drug, and subsequent tests confirmed the prediction that they would be as sensitive to the cytotoxic effect of MTX-HSA-791T/36 conjugate as the untreated 791T parental line. There were minor differences in colony formation at particular concentrations of conjugate, but the $IC_{50}$ values for parental cells and clones fell in the narrow range of 1.5 to 3.0 $ng\,ml^{-1}$ expressed in terms of MTX content. Extrapolated to the *in vivo* situation, these extremes are unlikely to be of practical significance in therapy. It is possible that these clones arose from cells which temporarily became drug-insensitive during the initial exposure but regained their susceptibility during subsequent clonal expansion, or that the threshold of effectiveness of MTX was not reached in the initial exposure.

The overall conclusion from these experiments is that cells escaping the effects of a cytotoxic drug-monoclonal antibody conjugate do not necessarily constitute truly resistant clones, but may be either incapable of further progressive growth (i.e. only short-term survivors) or long-term survivors susceptible to a further treatment with the same conjugate. This supports the design of protocols for *in vivo* therapy employing multiple doses of conjugate spread over an extended time period.

This work was supported by the Cancer Research Campaign, London, U.K. The skilled technical assistance of Mr D.G. Fox and Mr J. Lawry is gratefully acknowledged, and we are indebted to Dr R.A. Robins for advice on flow cytofluorometry. The 791T cell line was obtained from the U.S. Naval Biomedical Center, Oakland, Ca., U.S.A., by arrangement with Dr W.A. Nelson-Rees as part of a collaboration with Dr V.S. Byers.

### References

EMBLETON, M.J., GUNN, B., BYERS, V.S. & BALDWIN, R.W. (1981). Antitumour reactions of monoclonal antibody against a human osteogenic sarcoma cell line. *Br. J. Cancer*, **43**, 582.

EMBLETON, M.J., ROWLAND, G.F., SIMMONDS, R.G., JACOBS, E., MARSDEN, C.H. & BALDWIN, R.W. (1983). Selective cytotoxicity against human tumour cells by a vindesine-monoclonal antibody conjugate. *Br. J. Cancer*, **47**, 43.

GARNETT, M.C., EMBLETON, M.J., JACOBS, E. & BALDWIN, R.W. (1983). Preparation and properties of a drug-carrier-antibody conjugate showing selective antibody-directed cytotoxicity *in vitro*, *Int. J. Cancer*, **31**, 661.

GHOSE, T. & BLAIR, A.H. (1978). Antibody-linked cytotoxic agents in the treatment of cancer: current status and future prospects. *J. Natl. Cancer Inst.*, **61**, 657.

HARRAP, K.R., HILL, B.T., FURNESS, M.E. & HART, L.I. (1971). Sites of action of amethopterin: intrinsic and acquired drug resistance. *Ann. N.Y. Aca. Sci.*, **186**, 312.

KÖHLER, G. & MILSTEIN, C. (1975). Continuous cultures of fused cells secreting antibody of predefined specificity. *Nature (Lond.)*, **256**, 495.

PRICE, M.R., CAMPBELL, D.G., ROBINS, R.A. & BALDWIN, R.W. (1983). Characteristics of a cell surface antigen defined by an anti-human osteogenic sarcoma monoclonal antibody. *Eur. J. Cancer Clin. Oncol.*, **19**, 81.

PUCK, T.T., MARCUS, P.I. & CIECIURA, S.J. (1956). Clonal growth of mammalian cells *in vitro*, growth characteristics of colonies from single HeLa cells with or without a "feeder" layer. *J. Exp. Med.*, **103**, 273.

THORPE, P.E. & ROSS, W.C.J. (1982). The preparation and cytotoxic properties of antibody-toxin conjugates. *Immunol. Rev.*, **62**, 119.

TRELEAVEN, J.G., GIBSON, F.M., UGELSTAD, J., REMBAUM, A., PHILIP, T., CAINE, G.D. & KEMSHEAD, J.T. (1984). Removal of neuroblastoma cells from bone marrow with monoclonal antibodies conjugated to magnetic microspheres. *Lancet*, **i**, 70.